# Assays to Monitor Autophagy Progression in Cell Cultures

**DOI:** 10.3390/cells6030020

**Published:** 2017-07-07

**Authors:** Idil Orhon, Fulvio Reggiori

**Affiliations:** Department of Cell Biology, University of Groningen, University Medical Center Groningen, A. Deusinglaan 1, 9713 AV Groningen, The Netherlands; i.orhon@umcg.nl

**Keywords:** autophagy, autophagosome, phagophore, autophagic flux, measurement, assay, method

## Abstract

The vast number of implications of autophagy in multiple areas of life sciences and medicine has attracted the interest of numerous scientists that aim to unveil the role of this process in specific physiological and pathological contexts. Cell cultures are one of the most frequently used experimental setup for the investigation of autophagy. As a result, it is essential to assess this highly regulated molecular pathway with efficient and reliable methods. Each method has its own advantages and disadvantages. Here, we present a review summarizing the most established assays used to monitor autophagy induction and progression in cell cultures, in order to guide researchers in the selection of the most optimal solution for their experimental setup and design.

## 1. Introduction

Macroautophagy (hereafter simply referred to as autophagy) is an evolutionarily-conserved intracellular catabolic pathway that allows the turnover of macromolecules and organelles in eukaryotic cells [1]. This highly dynamic process is characterized by the formation of double-membrane vesicles called autophagosomes, which sequesters the cargo targeted for destruction before fusing with lysosomes to deliver their content for degradation. Autophagosome biogenesis starts with the formation of a membranous cistern known as the phagophore or isolation membrane, which expands and upon fusion of the two extremities through a fission event, leads to the generation of a double-membrane vesicle [2]. Complete autophagosomes fuse first with endosomes and, subsequently, with lysosomes to form amphisomes and autolysosomes, respectively [3]. Autophagy is regulated by the autophagy-related (*ATG*) genes and it can be non-selective or selective [4]. Non-selective, bulk types of autophagy are characterized by heterogeneous cargoes that appear to be randomly sequestered in the interior of autophagosomes. Selective types of autophagy, in contrast, involve the so-called autophagy receptors and lead to the exclusive enwrapping of specific structures into autophagomes.

Autophagy is an adaptive response to stress, but it is also involved in a multitude of other physiological processes, including intracellular quality control, prevention of cellular ageing, cell differentiation and development, cell death, stemness maintenance, and innate and adaptive immunity [5]. Consequently, impairments or defects in this pathway lead to severe pathologies, including specific types of cancer, neurodegeneration, and muscular dystrophies [6]. All these implications have attracted an enormous interest from the scientific community, as well as the pharmaceutical industry, in studying the regulation, mechanism, and functions of autophagy. As a result, it has become of primordial importance to monitor autophagic activity specifically, accurately, and reliably and, thus, to be able to determine changes of autophagic flux and eventually the blocked step when this pathway is impaired. The goal of this review is to summarize the established methods that are most frequently used to assess non-selective, bulk autophagy induction and progression, with the purpose of easy access to the detailed practical information that can be found in the literature (Table 1). The description of assays for the measurement of selective types of autophagy is out of the scopes of this review and detailed methods can be found elsewhere (e.g., [7,8,9,10,11]).

## 2. Materials and Methods

### 2.1. Methods for the Direct Measurement of Autophagic Activity

#### 2.1.1. The Turnover of Long-Lived Proteins

A direct method to measure autophagic flux is to monitor the degradation of a group of autophagosomal cargoes, e.g., the long-lived proteins [29,30]. This approach is based on the principle of radiolabeling long-lived proteins with radioactive amino acids, such as [^14^C]-leucine, [^3^H]-leucine, [^14^C]-valine, or [^35^S]-methionine [31], before inducing autophagy and measuring the release of radioactivity from the labeled proteins, which reflects an activity of this pathway. The involvement of the proteasome in the degradation of both short-lived and long-lived proteins is important. The turnover of short-lived proteins by the proteasome can be mostly excluded by discarding the culture medium after a chase of e.g., 4 h [12]. To rule out the contribution of the proteasome in the degradation of long-lived proteins from the assay, one option is to use proteasome inhibitors, such as MG132 [32]. The other is to perform the experiments in presence or absence of autophagy inhibitors such as 3-methyl adenine (3-MA), wortmannin, bafilomycin A1, chloroquine, NH_4_Cl, or leupeptin, to block lysosomal degradation and determine the non-autophagic protein degradation background [12] (Table 2). The detailed methodology for the execution of the long-lived protein degradation assay is explained in detail in the literature [12].

Long-lived protein degradation by autophagy can also be analyzed quantitatively using non-radioactive labeling with L-azidohomoalanine (AHA) [13,33]. Cells are conveniently pulse-chase labeled with this alanine analog as in the standard long-live protein degradation assay, before performing a click reaction in cells that have been fixed and permeabilized. The click reaction allows the cross-linking of the azide group of AHA with the alkyne group of an alkyne-tagged fluorescent dye. A change in fluorescence intensity in the cells, which can be measured using various techniques e.g., flow cytometry or immunofluorescent microscopy, indicates a variation in the activity of autophagy [14]. The major advantage of this technique is that it does not require a special room since not involving radioactive material. Its disadvantages are, firstly, the limitation in the selection of culturing media that can be used due to the AHA labeling that requires the absence of methionine and, secondly, 5% of the proteome only contains the N-terminal methionine and, consequently, is very poorly labeled with AHA.

Independently of the methods used to measure the turnover of long-lived proteins, our suggestion is to first employ more simple techniques to establish the experimental conditions and/or determine whether there is a change in the autophagic flux, before proceeding with this measurement.

#### 2.1.2. LDH Sequestration

Another method to directly assess the autophagic activity is to measure the sequestration of endogenous lactate dehydrogenase (LDH). LDH is a ubiquitous and abundant cytosolic enzyme that is non-selectively enwrapped into autophagosomes, and its sequestration can thereby be used as a measurement of autophagy. In particular, the measurement of the LDH pool in the cytosol versus the one that is sequestered allows a quantitative analysis of the autophagic flux. It is important to include lysosomal inhibitors in the samples to avoid the degradation of part of the sequestered LDH in lysosomes. After separating the sequestered and the non-sequestered fractions by centrifugation, the LDH amounts in the two fractions can be determined by either the enzymatic measurement of NADH decline or by LDH levels by western blot [16]. This technique was initially developed by Seglen and colleagues for isolated rat hepatocytes [34]. Recently, this assay has been adapted to mammalian cell cultures and validated by showing the accumulation of sequestered LDH upon treatment with various autophagy activators and lysosomal inhibitors such as vinblastine, chloroquine, rapamycin, and leupeptin/pepstatin (Table 2) [16,35].

Detailed practical reviews on the LDH sequestration assay have recently been published [15,16] (Table 1). As for the long-lived protein turnover assay, it is advised to use a more straightforward method to set the experimental conditions and/or establish whether there is a change in the autophagic flux before applying this method.

### 2.2. Methods for the Indirect Measurement of Autophagic Activity

#### 2.2.1. Western Blot-Based Assays

##### LC3 Lipidation 

Members of the microtubule-associated protein 1 light chain 3 (MAP1-LC3) protein family, the mammalian homologues of yeast Atg8, play an important role in autophagosome biogenesis and closure [36,37]. LC3 proteins are classified into the LC3 and GABARAP subfamilies, and the mostly-studied isoform is LC3B [38]. Members of the ATG4 cysteine protease family cleave the C-terminal amino acids of newly-synthesized LC3 proteins to expose a glycine residue, which can subsequently be conjugated to phosphatidylethanolamine (PE) upon autophagy induction, leading to their association to the internal and external surface of the expanding phagophores [39]. While the LC3 population on the surface of autophagosomes is released upon completion of these vesicles by ATG4 protease cleavage, the internal pool of LC3 is delivered and degraded in the lysosome together with the cargo. The lipidated form of LC3, also referred to as LC3-II, has an apparent molecular weight of 14–16 kDa on SDS-PAGE gels while non-lipidated LC3-I migrates around 16–18 kDa [36]. This difference in mobility, which is detectable by western blot, is frequently used as an indirect way to determine autophagosome formation through the measurement of LC3-II levels. It must be noted that the ratio between LC3-I and LC3-II can vary depending on both the examined cell type and the employed antibody because this latter could recognize preferentially one of the two forms of LC3. 

Analysis in the difference of the LC3-I/LC3-II ratio, however, will remain inconclusive without inhibition of the lysosomal degradative activity. Low levels of LC3-II could be due to an inhibition of autophagy or a high autophagic flux. Conversely, high levels of LC3-II could be caused by the impairment in autophagosome fusion with lysosomes or cargo degradation, or induction of autophagy. Pharmacologically blocking the proteolytic activity of lysosomes prevents the turnover of the LC3-II pool present in the autophagomes allowing the quantification of the total levels of LC3-II generated over the period of the experiment. As a result, the combinatorial evaluation of the same sample treated or not with a compound blocking lysosomal degradation, permits to determine whether there has been an induction or inhibition of the autophagic flux [29]. One of the most frequently employed drugs is Bafilomycin A1, an inhibitor of the lysosomal V-ATPase. Bafilomycin A1 provokes an increase in the lysosomal pH that blocks lysosomal degradation and, in specific cell types, also the fusion of autophagosomes with lysosomes. Other compounds such as protease inhibitors, e.g., a cocktail of pepstatin A, leupeptin and E-64d, or other pH modulators, such as chloroquine or NH_4_Cl, can also be employed (Table 2) [40,41].

In conclusion, the analysis of LC3 lipidation by Western blot is the go-to method to assess changes in autophagy in most cell lines. It is advised to perform this analysis in presence and absence of a lysosomal inhibitor. When possible, starving the cells for nutrients could provide a positive control, while the depletion of one of the ATG proteins could serve as the negative control. It is also important that when quantifying LC3-II, the LC3-II/loading control protein ratio is calculated rather than the LC3-II/LC3-I ratio since the latter might not correctly reflect changes in autophagy. One important note is that while LC3 was previously believed to be essential for autophagy, recent reports have shown that both starvation-induced bulk autophagy and PINK1/Parkin-dependent mitophagy do not require the members of the LC3 protein family [37,42]. Moreover, the autophagic flux can be maintained at high levels even at time periods when there was no LC3 flux under starvation conditions [42]. Altogether, these studies indicate that, under specific circumstances, LC3 may have a very limited value as a general marker protein for autophagy. 

##### SQSTM1/p62 Turnover

The turnover of SQSTM1/p62 is an alternative method widely used to monitor autophagic activity by Western blot. This protein is an autophagy receptor for various ubiquitinated cargoes [43]. SQSTM1/p62 binds specific cargo proteins through an ubiquitin-binding domain and simultaneously anchors them onto the LC3 pool present on the autophagosome interior, through an LC3 interacting region (LIR). These interactions allow the sequestration of targeted cargoes into nascent autophagosomes and subsequent lysosomal degradation. SQSTM1/p62 turnover can serve as a marker for autophagic activity in normal growing conditions in cells where this transport route works at basal level [44]. An induction of autophagy, however, increases the degradation of SQSTM1/p62 while a decrease or block of this pathway causes its accumulation. Identically, as for the LC3 Western blot analysis, the measurement of the autophagic flux using SQSTM1/p62 requires that the experiments are performed in the absence and presence of lysosomal inhibitors. 

An important aspect to consider when analyzing SQSTM1/p62 turnover is that the expression of this protein is transcriptionally regulated. It has been reported that under certain conditions, such as amino acid starvation, some cell lines have increased SQSTM1/p62 expression and, although autophagy is stimulated, the steady-state levels of SQSTM1/p62 remain unchanged because degradation is compensated by the de novo synthesis [18]. As a result, it is not possible to use the assessment of SQSTM1/p62 turnover by Western blot to measure the autophagic flux in all tissues. A way to employ this method also in cell types, where the steady-state levels of SQSTM1/p62 appear to vary upon autophagy induction, is to add cycloheximide in order to block the de novo synthesis or to perform a pulse-chase radiolabeling experiments followed by SQSTM1/p62 immuno-precipitation [18]. 

Several methodological reviews describing the more practical aspect of these Western blot analyses can be found in the literature [17,18,19] (Table 1). Overall, however, it is advised to analyze SQSTM1/p62 turnover in combination with LC3 lipidation levels for a firmer establishment of changes in the autophagic flux.

##### Post-Translational Modifications during Autophagy

Post-translational modifications (PTM) have a key role in the regulation of autophagy in both basal and stress-induced conditions, and several of them are regulated by the nutrient and energy state of the cell [45]. The most widely characterized PTM are amino acid phosphorylations by protein kinases, which are antagonized by the action of protein phosphatases [46].

The serine (Ser)/threonine kinase ULK1 and ULK2 have a redundant role, and they are both part of a complex that also includes ATG13, FIP200, and ATG101. In the presence of nutrients, this complex is associated with an active mTORC1 complex, which phosphorylates ULK1 at Ser638 and Ser758, ATG13 at Ser389, and AMBRA1 at Ser52 [45], and thereby inhibits the kinase activity of ULK1 and ULK2 and, ultimately, autophagy. Another inhibitory phosphorylation of ULK1 is maintained by the AKT kinase, which responds to insulin signaling, at Ser774 [47]. Upon nutrient depletion and consequent inhibition of mTORC1, dephosphorylation of ULK1 is followed by both autophosphorylation at Ser180 and ULK1-mediated phosphorylation of ATG13 at Ser318 and Ser203, FIP200 at Ser943, Ser986, and Ser1323, and AMBRA1 at Ser465 and Ser635 [48,49]. Another stimulus for autophagy activation is the decrease in the cellular energy levels, which are sensed by the AMPK kinase, which phosphorylates ULK1 at Ser317 and Ser777 triggering its activity [50]. AMPK also activates TSC2, which inhibits mTORC1 and thereby blocks the inhibitory phosphorylation of ULK1 [29]. All these PTM can be monitored by Western blot using phospho-specific antibodies, and their examination, singularly or in combination, indicates whether autophagy is induced or inhibited. While preparing samples, the presence of phosphatase inhibitors is essential to be able to detect these PTM. The activity of mTORC1 pathway and AMPK can also be used as an indirect method to examine autophagic activity by western blot, through the analysis of the phosphorylation status of specific downstream substrates of these kinases, such as 4EBP-1, RPS6KB/p70S6, or RPS6/S6 [51,52]. 

Another important PTM that can be monitored by Western blot are acetylation/deacetylation. This type of modification is often dictated by the availability of nutrients since the levels of acetyl co-enzyme A (AcCoA) drop when those become scarce. Deacetylation of specific proteins can, thus, be observed under conditions triggering autophagy such as caloric restriction [53]. General deacetylations can be quantified directly by either Western blot or immunofluorescence, with antibodies recognizing acetylated lysine residues [54]. Components of the ATG machinery can be directly acetylated/deacetylated, e.g., LC3 deacetylation, or their expression can get modulated through the deacetylation of transcription factors such as FOXO that are required for *ATG* gene expression [55]. The use of acetylation-specific antibodies but in this case on specific immunoprecipitated proteins, can provide an indication about autophagy progression. 

Detailed reviews on how to analyze deacetylayion and other PTM when monitoring autophagy are available [20,21] (Table 1). This approach, however, must not be the primary methods of choice to monitor variations in the autophagic flux since quite indirect. Detecting specific PTM might be valuable to point to the signaling pathway regulating the changes of autophagy that are being studied.

#### 2.2.2. Fluorescence Microscopy-Based Methods

Fluorescent microscopy allows a qualitative and quantitative analysis of the different step of autophagy through the examination of the subcellular distribution of either endogenous autophagosomal protein markers by immunofluorescence or ectopically-expressed fusion proteins by either immunofluorescence or live-cell imaging. When relying on the transfection of reporter proteins, the generation of stably-transfected cell lines permits obtaining a homogeneous protein expression with the advantage of decreasing the variability of signal intensity and size within a cell population. Consequently, stable transfected cell lines are especially suitable for automated microscopy-based measurements as well as a live-cell imaging [29]. The use of autophagy and lysosomal inhibitors allows to optimally control these experimental approaches as well.

Dyes labeling autophagosomal compartments such as Cyto-ID^®^ Green dye, can also be an option to visualize autophagic activity microscopically, especially in living cells or in cells that are difficult to be transfected. The major limitation of these dyes is their partial specificity but with the appropriate controls, they can be reliably used in high-content screens or difficult experimental settings [56,57].

##### Distribution of Autophagosomal Protein Markers

The members of the LC3 protein family are cytosolic and they change from a homogeneous cytoplasmic distribution into puncta upon autophagy induction as they become conjugated with autophagosomal membranes [36]. This difference in localization allows the quantification of autophagosomes within each individual cell. Since autophagosome fusion with lysosomes will ultimately lead to LC3 protein degradation, lysosomal inhibitors should be included in the experimental setup to determine the total number of autophagosomes that are formed over the time of the experiment. It must be noted that the number of LC3-positive puncta per cells in normal conditions can differ greatly, which may reflect different levels in basal autophagy [58]. Consequently, stimulation of autophagy does not always lead to a significant change in the number of LC3-positive puncta per cell.

Although all members of the ATG machinery localize to autophagosomes, the most studied one is LC3B because it is ubiquitously expressed and good antibodies, constructs, and stable cell lines are available as it was one of the first members of the LC3 protein family to be characterized. Since its expression levels can vary considerably from tissue to tissue, GFP-LC3 has been frequently used to surmount this limitation [36,59]. The sensitivity in GFP-LC3 detection, however, is variable from one cell type to another in culture conditions because of GFP quenching in low pH [29]. Therefore, alternative fluorophores such as mCherry, mRFP, and mTAgRFP, or protein tags detectable by immunofluorescence, are more ideal to probe LC3 [22]. Another important point of caution is that overexpressed GFP-LC3 can form puncta corresponding to aggregates rather than autophagosomes [60].

SQSTM1/p62 can also be used to monitor autophagy induction and flux. This autophagy receptor forms aggregates upon autophagy stimulation, which are delivered and degraded in lysosomes [61]. SQSTM1/p62 appears as discrete puncta when analyzed by immunofluorescence. Endogenous SQSTM1/p62 can be detected practically in all tissues because it is expressed ubiquitously. Although formation and accumulation of SQSTM1/p62 puncta can reveal an induction and a defect in autophagy, respectively, the additional use of lysosomal inhibitors allows estimating the autophagic flux. Several low and high throughput, fluorescent microscopy-based screens have exploited the SQSTM1/p62 puncta formation to identify autophagy modulators (e.g., [62,63,64,65,66]).

The analysis of the subcellular distribution of early autophagosomal protein markers such as double FYVE-containing protein 1 (DFCP1), WD-repeat domain phosphoinositide-interacting-1 (WIPI1) and WIPI2 also permits to specifically follow the induction of autophagosome biogenesis. These proteins are not abundant and consequently their localization is almost always examined by transfecting cells with plasmids expressing fusion proteins. DFCP1 is associated to the ER and through the binding of the PtdIns3P pool present on phagophores, relocalizes to punctuate structures corresponding to forming autophagomes upon autophagy stimulation [67]. This subcellular redistribution can be easily monitored and it is an indicator of autophagy induction. WIPI1 and WIPI2, in contrast, are cytoplasmic but they also associated to autophagosomal precursor structures through PtdIns3P binding [68]. Here as well, this easily detectable change in localization allows establishing whether autophagy has been triggered. It is important to note, however, that the autophagic flux and the effects of the autophagy stimuli are highly variable between cell types. Therefore, these fluorescent microscopy-based methods should be approached cautiously since some events maybe underrepresented depending on the time point of the experiment.

The localization of other ATG proteins, including ATG9A, ATG13, FIP200, ULK1, ATG16, and SYNTAXIN17, or organelle protein markers, such as lysosomal LAMP1, might also be employed to provide evidence for autophagic activity and crosstalk with other cellular pathways [29,69]. Examination of one or more specific marker proteins allows monitoring a specific stage of autophagy or when a defect is detected, determine the step that is blocked. Nonetheless, the advice is to use the fluorescent-base assays in combination with other methods to measure autophagy, since some of the mentioned marker proteins are also involved in other pathways.

##### Molecular Tandem Probes

A very important development in the field of autophagy was the generation of fluorescent-tagged probes to visually monitor the autophagy flux. The most frequently used one is LC3 fused with either mRFP-GFP or mCherry-GFP [22]. This probe generates a yellow signal when LC3 is associated to cytoplasmic autophagosomal precursor structures and vesicles. Upon fusion of autophagosomes with lysosomes, the low pH of these organelles quenches the GFP signal, which results in only the mRFP or mCherry fluorescent signal being detectable in the autolysosomes. The quantification of the yellow and red puncta consequently permits a quantitative measurement of both the induction and flux of autophagy [22].

Recently, a new fluorescent probe, i.e., GFP-LC3-RFP-LC3∆G, has been developed to monitor the autophagic flux [23]. This probe is cleaved by endogenous ATG4 proteases resulting in the formation of two fusion proteins, GFP-LC3 and RFP-LC3∆G. GFP-LC3 is partially degraded by autophagy while RFP-LC3∆G, a mutant form of LC3 that cannot be conjugated to PE, remains free in the cytosol as it is not a substrate of autophagosomes. The GFP/RFP signal ratio thus measures the autophagic activity [23]. 

Another recently-developed method to monitor the autophagic flux is the one based on the renilla luciferase-LC3 (RLuc-LC3) reporter construct [70]. Although not a tandem probe, degradation of RLuc-LC3, and the consequent decrease in the luminescence signal, allows monitoring of the autophagic flux. It is crucial to include the non-lipidable RLuc-LC3G120A construct in the experiments as a negative control.

#### 2.2.3. Electron Microscopy

Transmission electron microscopy (EM) has a central position in the field of autophagy as autophagosomes were first observed and described as large double-membrane vesicles sequestering cytoplasmic components by Christian de Duve in 1950s using this technique [71]. Opposite to the rest of the other methods commonly used to monitor autophagy, EM allows to directly count autophagosomes and autolysosomes [72,73]. The sequestration of specific substrates by autophagosomes, e.g., organelles or bacteria, can be distinctly analyzed and also quantified during the dynamic process of phagophore elongation and completion, and if cells are incubated with lysosomal inhibitors, the autophagosomal cargo can be promptly detected in the interior of lysosomes [24]. The correctness of the morphological observations depend highly on the procedure applied for sample preparation as for example, visualization of the double membrane of autophagosomes can exclusively be detected when cells are fixed using determined methods [74,75,76]. Various EM techniques have been applied to follow autophagy at the ultrastructural level, including transmission EM, immuno-EM, electron tomography, cryo-soft X-ray microscopy, and focused ion beam scanning electron microscopy (FIB-SEM) [77,78]. Detailed reviews describing the application of these techniques for the study of autophagy are available [9,10,26,36].

In most of the situations, however, an analysis of cells by conventional transmission EM in presence and absence of lysosomal inhibitors, is sufficient to determine whether there is an enhancement of the autophagic flux. Upon autophagy induction, the number of degradative compartments increases and as a result the quantification of the number of autolysosomes, lysosomes and amphisomes per cell section, provides a simple morphological readout [29]. Since it is very difficult to distinguish between autolysosomes, lysosomes, and amphisomes, even for an expert eye [74], it is much simpler to classify all these organelles together, as degradative compartments. One should keep in mind that in specific situations, the number of degradative organelles could change independently of an induction of autophagy. Although it is also possible to count autophagosomes, their transient appearance makes it very difficult to detect them when the fusion of autophagosomes and lysosomes are not pharmacologically blocked.

Ultrastructural approaches are time-consuming and, therefore, it is advised to use them to obtain firm conclusions on key experiments. It must be noted that the morphology of the cell is not well preserved under certain conditions and that the volume of the cytoplasm is reduced in cell types, such as stem cells, making the detection of autophagosomes and degradative compartments difficult.

#### 2.2.4. Flow Cytometry and Imaging Flow Cytometry

Both flow cytometry and imaging flow cytometry can be employed as a high-content analysis method to measure autophagic flux in living cells, especially if those do not adhere to surface, e.g., blood cells. While flow cytometry exclusively acquires an intensity reading for the entire signal in the cell, imaging flow cytometry, which is a combination of flow cytometry and microscopy, collects fluorescent images of the cells and as a result the location of the signal within the cell can be visualized and measured. The type of information acquired with these two approaches is consequently very different.

In cells expressing GFP-tagged LC3 or RLuc-LC3, induction of autophagy will lead to the transport of this protein into the lysosome, where GFP is quenched by protons and/or degraded. Therefore, a decrease in the total fluorescence or luminescence signal per cell, will reflect an autophagic activity [70,79,80,81,82,83]. The use of cells expressing tandem mRFP/mCherry-GFP-LC3 allows to more directly assess the autophagic flux because as explained above, permits the differentiation between autophagosomes and autolysosomes. To have a better signal to noise read-out, however, cell permeabilization with saponin permits the release of the cytosolic pool of LC3 leaving, intracellularly, the portion of this protein that is associated with autophagosomes [80]. Fixation and permeabilization of the cells also allows the staining and measurements of endogenous LC3, and an analysis of the autophagic flux can be obtained by treating, or not, cells with lysosomal inhibitors for the reason explained throughout this review [84]. Fluorescently-tagged autophagy substrates such as GFP-SQSTM1/p62 or GFP-NBR1, can also be used as protein markers for autophagic flux measurements by flow cytometry and imaging flow cytometry as their degradation provokes a significant decrease in the fluorescent signal [85,86].

The use of flow cytometry and imaging flow cytometry to measure the autophagic flux, however, is not optimal for all cell types. Cells in suspensions or with low adherence are more ideal as scrapings of adherent cells could have indirect stimulatory effects on autophagy, creating artifacts [29]. Nevertheless, these techniques can be very convenient for high-content screens or to differentiate the autophagic flux between different cell populations by combining the autophagy marker proteins with other ones [65,66,82]. Detailed reviews on the practical aspects of the flow cytometry- and imaging flow cytometry-based methods to measure autophagy are available [27,28] (Table 1).

## 3. Conclusions

With the exponentially-increasing medical interest for autophagy, there has been a great focus in the development and optimization of various techniques to measure autophagy progression, in particular for experiments realized with mammalian cells in culture. In addition to the methods described above (Table 1), there are multiple other approaches to assess autophagy, as well as non-canonical types of autophagy or chaperone-mediated autophagy. Those include immuno-histochemistry or degradative compartment isolation through subcellular fractionation. Upon adaptation, the methods described in this review are also applicable for the measurement of autophagy induction and flux in newly-emerging in vitro systems, such as 3D mammalian cell cultures and organoids [87].

It is important to note that most of the assays used to monitor autophagy involve indirect methods and/or marker proteins that often are also part of other cellular pathways [88,89,90]. It is, therefore, critical and necessary that more experimental approaches are used in parallel to obtain a firm conclusion whether there is a change in the autophagic flux. We hope that this review provides a helpful handbook to guide researchers in selecting a few of them for their studies in mammalian cell cultures.

## Figures and Tables

**Table 1:** Recent publications providing detailed practical protocols to measure autophagic activity following the methods presented in this review.

**I. Direct Measurement of Autophagic Activity**	Turnover of long-lived proteins	[12,13,14]
	LDH sequestration	[15,16]
**II. Indirect Measurement of Autophagic Activity**	Western blot-based assays (LC3 lipidation, SQSTM1/p62 turnover and post-translational modifications)	[17,18,19,20,21]
	Fluorescence microscopy-based methods (distribution of autophagosomal protein markers and molecular tandem probes)	[22,23]
	Electron microscopy	[24,25,26]
	Flow cytometry and imaging flow cytometry	[27,28]

**Table 2:** Most frequently employed autophagy and lysosomal inhibitors. Refer to [29] for further details.

Compound	Class	Effect
Bafilomycin A1	Lysosomal inhibitor	It increases the lysosomal pH
Chloroquine	Lysosomal inhibitor	It increases the lysosomal pH
Protease inhibitors (e.g., E-64d, pepstatin A and leupeptin)	Lysosomal inhibitor	They inhibit lysosomal proteases
NH_4_Cl	Lysosomal inhibitor	It increases the lysosomal pH
Wortmannin	Autophagy inhibitor	It inhibits autophagosome biogenesis
3-methyladenine	Autophagy inhibitor	It inhibits autophagosome biogenesis
